# On the Œconomy of the Liver

**Published:** 1807-11

**Authors:** 


*October* 2, 1807.

---

### *To the Editors of the Medical and Physical Journal.*

Gentlemen,

I Have just received the last Number of your valuable Journal, and have been considerably amused by the Speculations of your Correspondent Mr. Dawes, on the *Œconomy of the Liver.* This Gentleman seems anxious to refute the various suggestions of preceding physiologists, or as he denominates them, " The variety of hypotheses which have been formed, to account for the important function of the liver," by an hypotheses the most fanciful I ever heard started. To say, that because secretions in general are formed from arterial blood, the bile must also be secreted from arterial blood, is, in my opinion, much the same as saying, that, because the kidneys secrete urine, the testes should also secrete that fluid. The bile is a fluid so very different from every other animal secretion, that, I think it even more than probable, that it is a secretion from venous blood. But let us reason on the subject, and take facts along with us.

When we consider the quantity of blood, which must be necessary to nourish a viscus of the size of the liver, and know that this nourishment is derived solely from the arterial blood; it will be admitted as a fact, not an hypotheses, that there is not much more blood conveyed by the hepatic artery than is sufficient for this nourishment. Mr. Dawes's position derives no support from Dr. Saunders's experiment, for I know by experiments of my own, (which by the bye, I may trouble you with some day) that there is very little difference in the quantity of serum in blood drawn at the same time from an artery and a vein. Nay, I have found found in some instances, that blood drawn at the same time from the jugular vein and external carotid artery, exhibited but very little difference in the quantity of sernm. And surely Mr. Dawes will not contend, that the bl ood, in both instances, was *arterial.*

Mr. Dawes seems to convert the spleen to a strange use, namely, that of supplying the liver with arterial blood. The functions of the spleen have been longcanvassed, and no viscus in the human body has been made subservient to such a variety of purposes. Yet its functions are simple, and its use appears to me, abundantly evident. In it we see one of the wise provisions of Nature, it serves merely to regulate the functions of the stomach; not by furnishing arterial blood to the liver, but by regulating the quantity of blood necessary for the secretion of the gastric juice; and, as such, I would call it the reservoir of the stomach. When the stomach is empty, there is of course, no necessity for the secretion of the gastric juice in any quantity, therefore the blood is diverted, if I may so speak, from the stomach, by the vessels of the spleen, which has full room to expand and receive this blood, which is returned like the blood from the other viscera to the vena portæ. On the contrary, however, when the stomach is filled with food, it occupies a greater space; it presses upon the spleen, and prevents it from receiving any great quantity of blood; and as the blood then cannot circulate in the spleen, it passes on to the stomach in greater quantity, and thus furnishes the increased secretion of that liquor peculiar to the stomach, and so necessary for digestion.

The coronary artery may be stimulated by the food, but it is only from the splenic vessels it can derive the supply so much wanted for furnishing a supply of the gastric juice. And we must look to the generally increased circulation for the supply of bile. For from what is the fact, namely, that the spleen is always pressed upon when the stomach is distended, instead of there being an increased quantity of Mr. Dawes's *spleno-arterial blood,* to be *elicited* for the secretion of bile, we find that the quantity of blood, which is returned by the vessels of the spleen called veins, is much less at that time, than when the stomach is empty, and when there is so much increased action to *elicit* it.

I am, &c.

*Richmond, October* 5, 1807. MEDICUS.

